# Loaded Functional Strength Training versus Traditional Physical Therapy on Hip and Knee Extensors Strength and Function Walking Capacity in Children with Hemiplegic Cerebral Palsy: Randomized Comparative Study

**DOI:** 10.3390/children9070946

**Published:** 2022-06-24

**Authors:** Hanaa Mohsen Abd-Elfattah, Fairouz Hatem Ameen, Reham Alaa Elkalla, Sobhy M. Aly, Noha Ahmed Fouad Abd-Elrahman

**Affiliations:** 1 Department of Physical Therapy for Pediatrics and Pediatric Surgery, Faculty of Physical Therapy, Badr University in Cairo, Cairo 11829, Egypt; 2 Department of Basic Science, Faculty of Physical Therapy, Badr University in Cairo, Cairo 11829, Egypt; fairouz.hatem@yahoo.com; 3 Department of Physical Therapy for Surgery, Faculty of Physical Therapy, Badr University in Cairo, Cairo 11829, Egypt; reham.elkalla@buc.edu.eg; 4 Department of Biomechanics, Faculty of Physical Therapy, Cairo University, Cairo 12613, Egypt; drsobhyaly@gmail.com; 5 Department of Physical Therapy for Women’s Health, Faculty of Physical Therapy, Badr University in Cairo, Cairo 11829, Egypt; nona.bird.79@gmail.com

**Keywords:** cerebral palsy, hemiplegia, loaded functional strength training

## Abstract

Objective: This study’s objective was to see how loaded functional strengthening exercises using a plantigrade foot position and a shoe supporter affected muscle strength and walking ability in spastic hemiplegic children. Methods: Seventy-two children with spastic hemiplegic cerebral palsy, both sexes, aged ten to twelve years, were randomly assigned into two groups equal in number (control and intervention groups). The control group received a specially designed physical therapy program, whereas the intervention group received a loaded functional strengthening exercises program using a shoe supporter to maintain a plantigrade foot position. The training program was carried out for 60 min, three times per week for three consecutive months. All participants were evaluated both before and after the therapy program by using a Medical Commander Echo Manual Muscle Tester dynamometer to assess isometric muscle power of hip and knee extensors on the affected side. To assess functional walking capacity, a 6 min walking (6MWT) test was used. Results: Study groups were comparable with respect to all outcome measures at entry (*p* > 0.05). Within-group comparison showed significant improvements in all measured variables. Furthermore, between-group comparison revealed significantly greater improvements (*p* < 0.05) in hip and knee extensors strength as well as the functional walking capacity in favor of the intervention group. Conclusions: In all the analyzed variables, loaded functional strength exercises from the plantigrade foot position were found to be considerably more effective in the intervention group than in the control group.

## 1. Introduction

Cerebral palsy (CP) is defined as a group of disorders that permanently occur in the development of movement and posture, leading to activity limitations, which are attributed to non-progressive disturbances in the developing fetal brain or in the infant brain [1].

Children with CP exhibit neurodevelopmental abnormalities such as spasticity, contracture, decreased coordination, inadequate voluntary control, and muscular weakness [2]. Among these, muscular weakness is a significant motor impairment for children with CP. Muscle weakness is more prominent distally, and the hip extensors, knee extensors, and ankle dorsiflexors are weaker than their antagonists [3].

Weak muscles, rather than spasticity, are the most common cause of motor function deficits in children with CP, highlighting the importance of strength training for these children. Strength training must be customized for children with CP and should focus on progressive increases in the load and intensity of the strengthening program, aiming to develop more strength gains than those seen during normal growth and development. These types of exercises, where there is a continuous increase in the load, are known as progressive resistive exercises, where muscles are overloaded manually, mechanically, or by weight-bearing [4].

Children with poor muscle function affecting the lower extremities usually have abnormal weight-bearing; they might not be able to push down through the whole foot while standing up or during the stance phase. They may also suffer the inability to balance and may exhibit propulsion defects, which might be due to lack of experience in weight-bearing through the feet in propulsion during standing and walking when they were infants. Another reason may be the early development of calf muscle contractures [5].

Most daily living activities follow a closed-kinetic chain that involves multiple-joint movements. Therefore, task-oriented functional strengthening programs including a closed-kinetic multi-joint approach would be more effective in the treatment of CP children, as this approach produces more significant outcomes regarding functional strength training with progressive resistive exercises [6]. 

In children with CP, studies have highlighted the benefits of strength training and the relationship between muscular power and exercise. Strength training improves muscle strength, flexibility, posture, and balance in CP patients. It also enhances functional activities such as walking and running by increasing daily exercise levels [7,8]. The key features of successful strengthening protocols include progressive overload; specificity in terms of muscle group, muscle action, and energy systems used, as well as velocity of contraction; delivery of sufficient exercise volume and load intensity to reach training goals and sufficient frequency of loading; provision of adequate rest periods for recovery; selection of appropriate form of resistance; and the design of appropriate training variation and periodization to maximize muscle adaptation [9].

Gains in strength are usually translated into more meaningful gains in functional motor performance when strength training is administered in the form of functional-oriented, closed-kinetic-chain exercises. In this case, weight is raised and lowered over the feet by concentric and eccentric action of the lower limb muscles, which mimics many activities involving the lower limbs, such as sit-to-stand and walking [10].

Although some evidence suggests that loaded functional strength training is increasingly being applied in the treatment of children with CP, there is a paucity of experimental evidence on its efficacy in the plantigrade foot position with a shoe supporter. As a result, the purpose of this research was to see how loaded functional strength exercises in a plantigrade foot position alter hip and knee extensors strength, as well as walking ability, in children with hemiplegic CP.

## 2. Materials and Methods

### 2.1. Study Design

A single-masked clinical trial was conducted at the Outpatient Physical Therapy Clinic of Faculty of Physical Therapy, Badr University, Cairo, Egypt. The current study’s clinical trial registration number is NCT 05068596. The Institutional Review Board of Cairo University’s Faculty of Physical Therapy approved this study, P.T.REC/012/002853, according to the requirements defined in the most recent edition of the Declaration of Helsinki code of ethics. Prior to data collection, the participation of children was mandated by having their parents or guardians sign an agreement.

### 2.2. Participants

**Figure 1:**
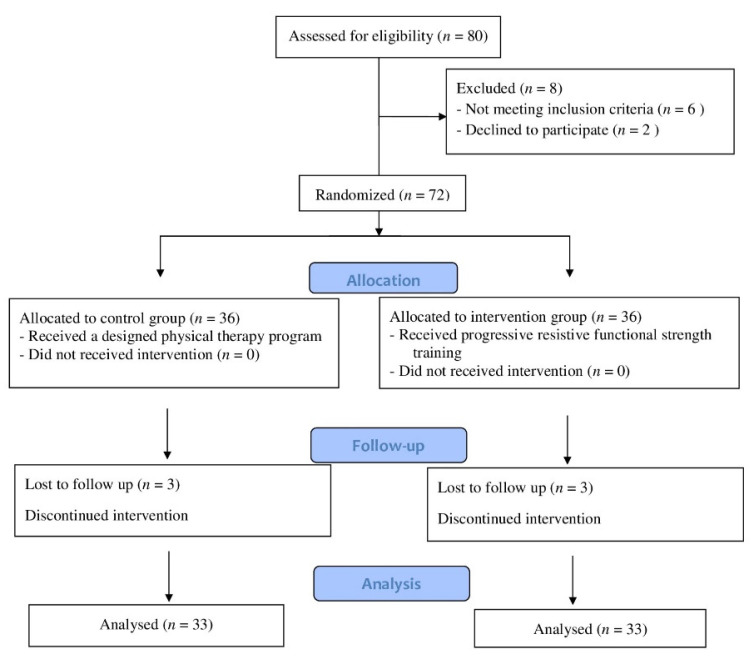
Children’s consort flow diagram.

Figure 1 depicts the flowchart for participant enrollment and engagement. Out of 80 children who were examined for eligibility, 72 fulfilled the inclusion criteria and were allocated randomly to the control and intervention groups. Six children were excluded due to a lack of follow-up (three children in the control group and three in the intervention group). The treatment program was completed by 66 children. Participants in this study ranged in age from ten to twelve years old, and both sexes were recruited. The inclusive criteria included hemiplegic CP children, verified by magnetic resonance imaging obtained from patient medical files, and mild spasticity of the lower limb according to Modified Ashworth Scale (MAS) grade 1 to 1+ [11]. According to the gross motor function classification scale (GMFCS), the participants were classified into levels I–II [12]. The children needed to be able to extend the knee from 90 to 45 degrees or more in a sitting position, with full passive range of motion in a supine position, and be able to flex the knee to 90 degrees in a prone position without simultaneous hip flexion. The children needed to have the cognitive abilities to comprehend and obey instructions. The exclusion criteria included 1—congenital defects of the lower extremities, 2—scar, 3—persistence contracture, 4—spinal or extremity anomaly, 5—visual or pulmonary problems, and 6—rhizotomy, or injection of botulinum toxin into the lower limb muscles during the previous 6 months.

### 2.3. Sample Size

Prior to the investigation, the sample size was calculated using G*POWER statistical software (version 3.1.9.2; Franz Faul, Universitat Kiel, Kiel, Germany) (MANOVA with F tests). The means of the 6MWT index were estimated using data from preparatory pilot research, which included 10 children who were randomly allocated to one of two treatment groups (5 children each). The acceptable sample size for this investigation was not less than 60 children, according to repeated measures, within–between interaction = 0.05, β = 0.2, Pillai V = 0.1, and effect size = 0.37. Then, to account for probable dropout rates, 72 children were allocated.

### 2.4. Randomization

For this study, 72 children with hemiplegic CP were enrolled. Following the baseline measurements, online Graph Pad software (GraphPad Software Inc, Graphpad Holdings, LLC, San Diego, CA, USA) was used to randomly assign 36 children to one of two groups: control (received the designed physical therapy treatment) or intervention (received loaded functional strength exercises in the plantigrade foot position). The allocation was performed by an impartial person who was not aware of the study protocol and was not in control of the trial in any other way. To ensure that children in both groups were evenly distributed, subjects were stratified by age and sex. The distribution of participants was kept hidden from all children, relatives, and researchers ultimately responsible for evaluations.

### 2.5. Outcome Measures

#### 2.5.1. Isometric Muscle Strength Measurements/Muscle Strength Dynamometry

The JTECH Medical Commander Echo Manual (MN022, Chester Springs, PA, USA) was used to measure isometric muscle strength, which was measured in kilograms. The handheld dynamometer is an objective and reliable device used to measure muscular action in a non-complicated method [13].

The dynamometer was stabilized by the therapist while the child pressed as much as possible against the dynamometer for a 3 s period, during which the peak force (in kilograms) was measured. The average of three repetitions was recorded for analysis [14].

#### 2.5.2. Functional Walking Capacity

The six-minute walk test (6MWT) was used to assess functional capacity, which is a submaximal quantitative evaluation of functional exercise capacity and the ability to perform physical activities used in daily living [15]. The child walked on an obstacle-free rectangular pathway, as mentioned in the regulations of the American Thoracic Society (2002) [16]. The therapist walked behind the child as near as possible to keep them safe without causing interruption, and used a stopwatch to measure the actual distance covered in 6 min [17].

### 2.6. Intervention

Children in the control group received a designed physical therapy program, which consisted of stretching for the lower extremity muscles, namely hip flexors and adductors, hamstrings, and calf muscles; and strengthening exercises for core muscles, hip extensors, flexors and abductors, internal and external rotators of the hip, flexors and extensors of the knee, ankle dorsi flexors, kneeling exercises, and standing and gait training.

**Figure 2:**
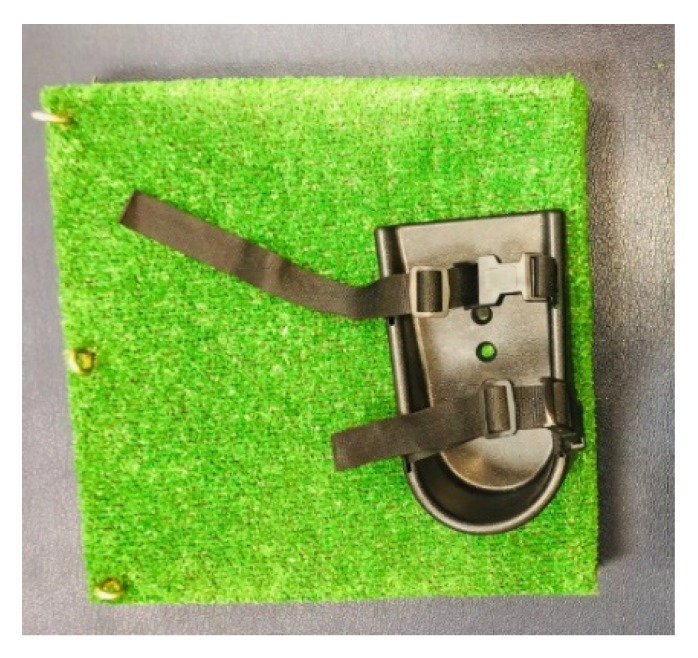
Shoe supporter.

Children in the intervention group were given loaded functional strength training while wearing the shoe supporter, which prevented them from plantar flexing and rising on their toes. The shoe supporter simply mounted onto a wooden footplate structure with the included nuts and bolts. The standard medium shoe supporters were 7.25 cm long, 3.25 cm wide at the heel, and 3.75 cm wide at the toe. They had a robust ABS plastic construction and adjustable length straps, with pinch buckle clips. The contoured sides followed the contours of the foot. They were suitable for use with either bare feet or shoes (Figure 2).

**Figure 3:**
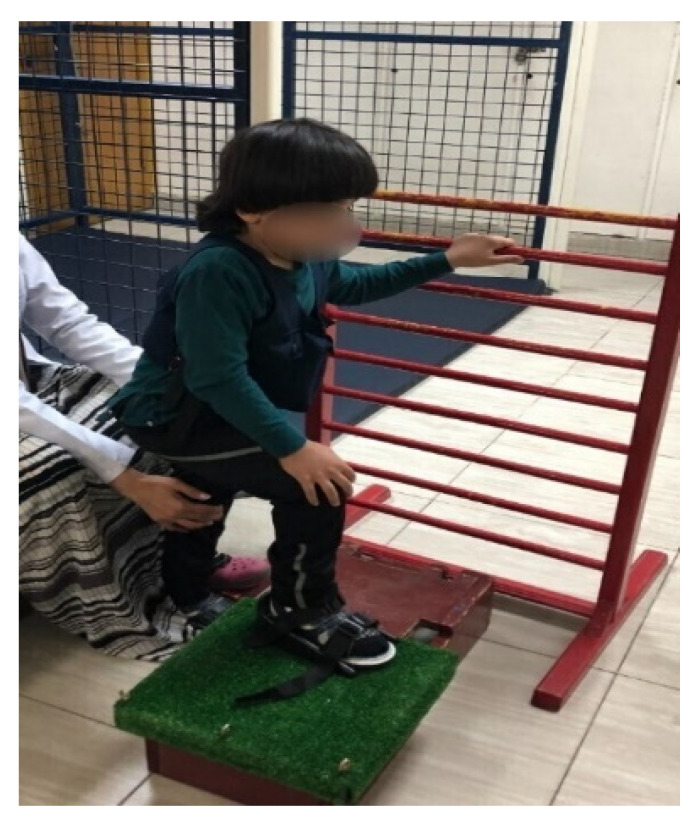
Forward step-up exercise.

Loaded functional strength training was carried out with resisted forward step-ups, lateral step-ups, squats, sit-to-stand, and stoop-and-recover exercises (Figure 3). The exercises were carried out in a circuit fashion. A customized weight vest was used to give resistance. The weight vest had a weight pocket bearing different loads, ranging from 0.25 to 6 kg. The weight vest’s load was evenly distributed anteriorly, posteriorly, and to the sides [6]. The weights were added into the pockets of the body vests based on the individual eight-repetition maximum (8 RM) test [18], according to gross motor function classification system levels I and II. We estimated 8 RM to be around 35% and 30% of the child’s total weight [4]. The load was gradually raised to match the child’s strength until all tasks were practiced with a maximum of 75% of 8 RM (Table 1). Each session was initiated with a 10 min warm-up period, including stretching of the major muscles and muscle groups, and terminated with a 10 min cool-down period in the form of aerobic exercises. Both groups received their training program for 60 min with a rest interval of 2 min between different types of exercises, with a frequency of 3 days/weeks for a duration of 12 weeks.

## 3. Results

### 3.1. Statistical Analysis

A unpaired *t*-test was conducted for comparison of subjects’ characteristics between groups. A chi-squared test was conducted for comparison of sex, spasticity grades, and GMFCS distribution between groups. The Shapiro–Wilk test was used to ensure that the data were normally distributed. To test the homogeneity of variances between groups, Levene’s test for homogeneity of variances was used. To compare within- and between-group effects on hip and knee extensor strength and 6MWD, a mixed-model MANOVA was used. For multiple comparisons, post hoc tests with the Bonferroni correction were conducted. For all statistical tests, the level of significance was set to *p* < 0.05. Data management and analysis were performed using SPSS version 25 for Windows for all statistical analysis (IBM SPSS, Chicago, IL, USA).

### 3.2. Subject Characteristics

Sixty-six children participated in this study. No significant differences were found in subjects’ age, weight, height, sex, spasticity grade, or GMFCS distribution between groups (*p* > 0.05) (Table 2).

### 3.3. Effect of Treatment on Hip and Knee Extensor Strength and 6MWD

Treatment and time had a significant interaction (F = 72.51, *p* = 0.001). Time had a significant main effect (F = 1170.73, *p* = 0.001). Treatment had a significant main effect (F = 73.84, *p* = 0.001).

There was a significant increase in hip and knee extensor strength and 6MWD of the control and intervention groups post-treatment compared with that pre-treatment (*p* < 0.001). The percent change in hip and knee extensor strength and 6MWD of the control group was 51.24%, 34.34%, and 9.69%, respectively, while those of the intervention group were 61.86%, 45.63%, and 20.09%, respectively (Table 3).

Pre-treatment, there was no significant difference between groups (*p* > 0.05). There was a statistically significant increase in hip and knee extensor strength and 6MWD in the intervention group compared with that of the control group post-treatment (*p* < 0.01) (Table 3).

## 4. Discussion

The purpose of this study was to determine how loaded functional strength training with a shoe supporter affects hip and knee extensor strength and walking abilities in children with spastic hemiplegic CP. After 12 successive weeks of applying the strength training program, the intervention group’s mean values of hip and knee extensor strength were significantly higher than in the control group, in addition to a significant increase in the functional walking capacity. This agrees with the findings of a former study performed on adolescents with CP, which showed a direct correlation between knee extensor strength and gross motor skill, proving that enhancement in muscular strength is closely related to improvements in walking performance and functional capacities [19].

Loaded functional strength training was used to strengthen the lower limbs in children with CP—with affecting spasticity—and to improve the functional motor skills, including gait performance, in CP children. Strength training suits older children and adolescents more than younger children as it requires maximum performance and the ability to perform compound activities [4,19,20], which supports the results of our study, namely the revealed significant effect of a loaded strength training program on hip and knee extensor muscles and on functional walking capacity.

The significant improvements in walking abilities reported in the intervention group could have been due to the increase in hip and knee extensor muscle strength, which is a determinant in acquiring an upright posture. This was consistent with the findings of Lowes et al., who found a direct relation between the strengthening of the muscles and motor functional abilities. Increasing the knee extensor strength in CP children has a strong correlation with functional motor abilities such as walking and standing. The stronger the knee muscles, the better the performance of the functional motor skills and the longer the duration of walking. Stronger knee muscles also preserve energy and enhance walking at a quicker pace [21].

Strength training, especially for the quadriceps femoris muscle, counteracts the hamstring muscle as it acts to decelerate the thigh during gait in the later swing phase, leading to improvement in knee extension and lengthening of the stride, along with minimizing the angle of flexion in the terminal swing phase of the gait cycle. There is also a significant improvement in the positioning of the foot prior to the initial contact with the ground. Furthermore, the force production in the quadriceps femoris muscles increases, resulting in spontaneous improvements in the gait performance of CP children [22].

As for the functional sit-to-stand daily activities and walking, which greatly depend on the gluteus maximus muscle, observable improvements were recorded regarding better coordination required for the body weight to be shifted onto the feet along with upward movements during the vertical phase of the gait. During the gait cycle, the gluteus maximus functions to control the pelvis, preventing the trunk from propelling forward during heel strike [23].

Regarding the post-treatment results of the intervention group, the development found in motor performance might be related to closed-kinetic-chain exercises, which proved to be more functional and appropriate for the assessment and rehabilitation of lower extremity performance than open-kinetic-chain exercises. This agrees with the findings of Blundell et al. [10], who proved that weight-bearing exercises represented in closed-kinetic chains were more related to functional motor activities, especially those involving ascending and descending stairs and walking, where the force and power induced by the lower limb muscles are directly related to the functional activity targeted by the training program.

Higher statistically significant differences in the isometric muscle strength of the lower extremity in the intervention group compared with the control group might be related to the plantigrade foot position achieved using the shoe supporters, which allowed the CP children to receive loaded functional strength training along with the weight-bearing exercise or closed-kinetic-chain and balance exercises. The supporter restricts the foot so that no plantarflexion or tiptoe rise occurs (which is a common problem in CP children with hemiplegia), giving the children the opportunity to focus on increasing the strength and motor control of the flexor and extensor muscles. This is in accordance with the findings of a study by Carr and Shepherd, which highlighted that balance activities and standing-up training in weight-bearing positions can be more effective using a shoe fixed to a flat surface, where the feet are retained with heels on the ground. This allows the children to understand the purpose of pushing down through the feet, performing hip and knee flexion and extension, and positioning the ankle with more precision over the feet [24].

Children with hemiplegic CP cannot bear weight and push down through the whole foot in functional activities such as sit-to-stand and forward and lateral step-up as a result of false weight-bearing in these propulsive actions during infancy. This is supported by another study that stated that sitting with the feet on the floor requires early training in infancy to enable balance through the lower limbs while seated and standing. The lower extremities play a major role in enhancing body balance. Therefore, it is critical to ensure efficient active elongation and force-generation of the lower-extremity muscles, along with boosting precise foot alignment during weight-bearing activities. However, such activities should be reinforced early in infancy, as neglecting them might lead to abnormal distribution of force on the plantar foot surface, leading to decreased balance and alignment over time [25].

Having a child wear a weighted vest during activities provides tactile and proprioceptive input, both of which are critical components of sensory integration, and provides load during progressive functional strength training for the muscles of the lower extremity. In addition, the use of this vest overcomes the necessity of having a CP child carry a heavy load and trying to balance with it to perform resistive exercises [26].

Our research has some apparent limitations. The absence of follow-up following the intervention was one of the current study’s crucial limitations. We assessed hip and knee extensors only, without measuring the remaining lower limb muscles. Therefore, it is advisable for upcoming research to include all the lower extremity muscles and to perform analyses on other types of CP and neurodevelopmental disorders. Evaluations of the influence of the same program on the different levels of engagement in daily living activities should be undertaken to gain further insight into its effect on all levels of the International Classification of Functioning, Disability, and Health, and the different impairment and activity limitations, as well as participation restriction.

## 5. Conclusions

The results of the current study suggested that both functional strength training and conventional physical therapies have the potential to produce significant improvements in hip and knee extensor strength and functional walking capacity in children with hemiplegic cerebral palsy. Moreover, conventional physical therapy was considered to be as effective but to a lesser extent than functional strength training. Loaded multi-joint functional resisted exercises (sit-to-stand, half-knee rise, and step-up) were designed to incorporate the fundamental tasks required for daily living performance during walking, stair climbing and general mobility.

## Figures and Tables

**Table 1:** Weekly training and timing of the 8 RM.

Week	Goal	Load
1–2	Getting child oriented with training program. Explaining different exercises to children. Determining initial starting position for different exercises. Practicing exercises in correct pattern.	BW
3–4	Determining initial starting position for different exercises. Practicing exercises in correct pattern.	5–10% BW
5	Gradually building up training intensity.	50% of 8RM
6	Gradually increasing training intensity.	60% of 8RM
7–12	Developing strength training.	75% of 8RM

**Table 2:** Participant characteristics.

Parameter	Control Group	Intervention Group	*p* Value
Age (years), Mean ± SD	11.11 ± 0.63	11.31 ± 0.62	0.18 ^a^
Weight (kg), Mean ± SD	37.12 ± 2.83	38.31 ± 3.93	0.14 ^a^
Height (cm), Mean ± SD	143.41 ± 4.21	142.55 ± 4.1	0.38 ^a^
Sex, *n* (%)			
Girls	13 (39.4%)	11 (33.3%)	0.61 ^b^
Boys	20 (60.6%)	22 (66.7%)	
Spasticity grades, *n* (%)			
Grade I	10 (30.3%)	8 (24.2%)	0.58 ^b^
Grade I+	23 (69.7%)	25 (75.8%)	
GMFCS, *n* (%)			
Level I	13 (39.4%)	10 (30.3%)	0.43 ^b^
Level II	20 (60.6%)	23 (69.7%)	

SD, standard deviation; *p* value, level of significance; ^a^, *t* test; ^b^, chi square test.

**Table 3:** Mean values of hip and knee extensors strength and 6MWD pre- and post-treatment of control and intervention groups.

Parameter	Control Group	Intervention Group	MD	*p* Value
	Mean ± SD	Mean ± SD		
Hip extensor strength (kg)				
Pre-treatment	3.22 ± 0.54	3.33 ± 0.65	−0.11	0.44 ^NS^
Post-treatment	4.87 ± 0.66	5.39 ± 0.67	−0.52	0.002 ^S^
MD (% of change)	−1.65 (51.24%)	−2.06 (61.86%)		
	*p* = 0.001	*p* = 0.001		
Knee extensor strength (kg)				
Pre-treatment	3.96 ± 0.56	4.12 ± 0.58	−0.16	0.25 ^NS^
Post-treatment	5.32 ± 0.61	6 ± 0.71	−0.68	0.001 ^S^
MD (% of change)	−1.36 (34.34)	−1.88 (45.63)		
	*p* = 0.001	*p* = 0.001		
6MWD (m)				
Pre-treatment	274.69 ± 5.98	276.66 ± 7.97	−1.97	0.26 ^NS^
Post-treatment	301.3 ± 3.73	332.24 ± 6.59	−30.94	0.001 ^S^
MD (% of change)	−26.61 (9.69%)	−55.58 (20.09%)		
	*p* = 0.001	*p* = 0.001		

SD, standard deviation; MD, mean difference; *p*-value, probability value, ^NS^ *p* > 0.05 = non-significant, ^S^ *p* < 0.05 = significant.
